# When does population growth pay off? A case study of suburban land consumption to assess the Lower Austrian infrastructural cost calculator

**DOI:** 10.1007/s10901-018-09639-7

**Published:** 2018-12-28

**Authors:** Alois Humer, Raphael Sedlitzky, David Brunner

**Affiliations:** 1 Department of Built Environment, Aalto University, Otakaari 4, 02150 Espoo, Finland; 2 Department of Geography and Regional Research, University of Vienna, Universitaetsstrasse 7/5, 1010 Vienna, Austria

**Keywords:** Density, Infrastructure, Land use, Local, Municipality, Public finance, Residential area, Settlement development, Spatial planning instrument

## Abstract

‘To increase the number of inhabitants’ is a commonly stated top objective in municipal strategies across European countries. Not differently in Austria, local policy follows a logic of growth as financial tax and redistribution systems reward according to population figures; but is demographic growth necessarily financially beneficial for a municipality, irrespective of the type of land use changes, and potentially urban sprawl, that it triggers? The Federal State of Lower Austria offers to its municipalities a strategic online planning tool to pre-assess eventual municipal infrastructural costs and tax revenues that would come with certain population increase. This study tests the Lower Austrian *infrastructural cost calculator* and, in so doing, seeks to add a spatial perspective to an otherwise oversimplified financial calculation of planning for growth. The case study municipality of Michelhausen formulated an ambitious objective of 25% population growth (+ 700 inhabitants) within a few years in its local development strategy, to be realised by enlarging a rural settlement area. The study will assess five possible alternatives of settlement enlargement with varying housing types for their municipal financial consequences. In conducting this case study, the *infrastructural cost calculator*, a strategic planning tool offered by the federal planning authority of Lower Austria to their municipalities, will be assessed for its current potential as well as possible enhancement as strategic planning instrument to support municipalities in financial questions when developing building land. Normative lessons drawn from the whole exercise directly address actors and decision-makers in local and regional planning context in Lower Austria. The study ends with a short outlook of possible learnings and transfer into other national and international planning practice contexts.

## Introduction: a local public perspective on land use, housing, infrastructure and finances

Regardless of the municipality’s settlement enlargement or densification potential, size, regional rank or connectivity, past or expected economic situation, the development objective is quite always ‘growth’. While densification oriented growth is widely acknowledged in urban planning contexts for environmental sustainability concerns (Sivam et al. 2012, p. 475), rural municipalities especially perpetuate traditional settlement patterns characterised by low density, detached housing types. Awareness and reflection is often lacking of what that means for future local public finances and infrastructure provision among policy and planning decision makers, or at least long-term financial consequences are ignored. The lack of acknowledgement is thereby mutual. While at times spatial planning objectives are unclear about financial consequences, fiscal policies do not take into account spatial impacts. For example, Dekel (1995, p. 935) criticises how “[c]onventional fiscal impact analysis has failed to address adequately the spatial dimensions of development alternatives, notably the costs associated with housing density.” We know from literature that the form of settlement development, i.e. how dense or sprawled it is, frequently has significant impact on public infrastructural costs (Ewing and Hamidi 2015, p. 422f). This contribution is motivated by the oversimplified but frequently used formula in local planning practice that growth—in terms of settlement enlargement and increase of inhabitants—automatically results in a positive outcome for a municipality, also financially. The federal planning authority of Lower Austria has developed a free online tool for municipalities and potential other actors in local land use planning to estimate expected financial costs and benefits that arise from certain, envisaged population growth. This study will by example draw on the planning objectives of one rural municipality, Michelhausen, in order to assess the usefulness and potential of the so-called *infrastructural cost calculator* as a strategic background instrument for local land use planning. The exercise will unfold whether the calculating tool can assist in answering the analytical question: ‘What short-term and long-term financial impact does it have on a municipality, where and how new inhabitants are being settled?’ The exercise will include a time-perspective into local public financial issues of settlement enlargement into alternative future development. The paper concludes with some normative lessons for spatial planning on the intersection of strategic planning instruments and local land use plans, informing planning practitioners and policy makers in Lower Austria, and potentially beyond.^1^

## Background: everyday land consumption, urban sprawl, and local planning practice

We regard a focus on financial efficiency of planning decisions as relevant because it can serve as a transparent argument in capturing the effects of urban sprawl, particularly in small, suburban municipalities. Principal components of urban sprawl are the density of settlement and the lack of land use mix (Hamidi et al. 2015). The many single land consumption activities like in Michelhausen, as we will investigate in course of this study, sum up to an average of currently (2015–2017) 12.9 ha/day in Austria. This is a remarkable decrease compared to the last decade, where yearly land take varied around 20 ha/day; but it still exceeds the officially formulated sustainability target of 2.5 ha/day by 5-times. Residential functions account for a share of close to 40% and so are, together with industrial use, the main purpose of land consumption (Umweltbundesamt undated). The major part of land consumption is taking place in suburban areas. The regional population and settlement trends are forecasted to continue and intensify. While several peripheral regions will face ageing population and outmigration, immigration pressure into urban areas and suburban hinterlands will increase. A particular concern is the ratio and its development over time between land consumption and number of inhabitants. For a long period, land consumption per capita increased overproportionally. At least, Statistik Austria (Statistik Austria undated a) reports a recent turning point since 2015, from which on the annual growth of population is less than the annual growth of land consumption. Still, forecasts published by ÖROK assume, under currently continuing situation of population increase, internal migration patterns and economic activities, an increase of land consumption of circa 17 ha/day by 2060. In spatial terms, suburban and semi-peripheral areas with low density and dispersed settlement structure will continuously account for major parts of future land consumption. (ÖROK 2015, 2017). Making efficient future land use a top priority in planning practice as well as mainstream it in other relevant sector policies is the core of a recent mutually agreed recommendation by the legal planning authorities in Austria (ÖROK 2017). This is in line with comparable other initiatives in Europe and, not least, is in line with the UN Sustainable development goals 11 and 15 (REFINA 2007–2012; SURFACE 2017–2020). While the awareness is and commitment is obviously rising on upper, strategic levels, it will nevertheless remain a decisive part with the actors on local level, to indeed develop land more efficiently in the future.

It is important to state that planning for growth is not a wrong strategy per se, but a necessity in most metropolitan areas in Europe including their suburban hinterland as a way of coping with current development trends of urbanisation, socio-demographic changes, economic dynamics and transportation improvements. However, growth is not a straightforward prospect when aiming for sustainable development and avoiding disproportionate land consumption. Despite the lack of regional cooperation, municipalities of various sizes and densities should question ‘where’ and ‘how’ spatial growth can be planned for. In terms of the ‘where’, we can distinguish two ‘ideal types’ of settlement growth, of which the first one is achieving higher densities within the current settlement boundaries—by rebuilding or filling up empty plots—and the second one is enlarging the current settlement with new building land on the outskirts. From a sustainability perspective, settlement development close to the first ideal type is a high planning objective, not least because of the more efficient consumption of land (Siedentop and Kausch 2004), especially in a rural land use context (Lange et al. 2015, p. 694). After locating possible development zones inside or outside the current settlement boundaries, various housing types, of ‘how’ to build or rebuild the land may result in very different settlement patterns, levels of infrastructure and living environments. The lowest density housing type—single-family houses—is still the most popular in Austria’s countryside. While semi-dense types like townhouses or row houses are present in bigger suburban agglomerations, multi-storey houses are in practice reserved for urban areas or some prominent traffic nodes in the suburbs. (Fassmann et al. 2009; Helbich and Leitner 2009). A study by Gruber-Rotheneder et al. (2012) on how to successfully increase the number of inhabitants shows a definitive 94% preference among mayors of over 200 municipalities in Vienna’s suburbs in favour of the single-family housing type. Likewise, a growth in the number of inhabitants is overwhelmingly considered positive (around 70%) and associated with additional public financial revenues and more liveable local life. The most commonly mentioned downside of population gain is increased traffic volume and eventual difficulties in integrating newcomers into the local community, whereas mayors commonly not consider financial burdens as a possible threat.

The mainstream instruments and practices within European spatial planning systems are deeply rooted in a planning culture that follows a growth logic, especially when it comes to the local level. Wiechmann and Bontje (2015), who confirm a primacy of the growth logic, see the reason in the genesis of planning systems and professions over the past decades. They situate the problem in the wider historical context. Today’s spatial planning systems were developed as European states were experiencing growth in major fields of population, economy and life standards in the second half of the 20th century. Adding to this the planning culture of growth is fuelled by a general administrative-political state organisation in European countries such as Austria that redistributes federal tax revenue at the local level according to the number of inhabitants. Inter-municipal policy cooperation is therefore unpopular when it comes to managing population increase or follow regional planning attempts in general (Fassmann and Humer 2013; Mönnich 2005; Reimer 2013, p. 4667).

## Case study: testing the Lower Austrian infrastructural cost calculator in a real planning practice case

In assessing the usefulness and potential of the Lower Austrian infrastructural cost calculator, we employ the tool to a real case study. Within this section, we introduce the context of land use planning in Lower Austria and present the planning tool of interest. We then use it to run alternative calculations of financial costs and benefits in the case of land consumption in the municipality of Michelhausen. We base the calculation on objectives given in the current local planning documents and respect the situative settlement structure. Learning from this extended test, we will draw conclusions concerning the planning tool itself in the last main section of the paper.

### Empirical context: land use planning and local public finances in Lower Austria

Spatial planning in Austria is governed at the local level. The constitution assures municipalities a self-governmental right for this field of policy. In practice, spatial planning is widely in hands of the roughly 2100 municipalities, the possibility for cooperation between municipalities however differs from state to state.^2^ In the case of the federal state of Lower Austria, municipalities are obliged to issue two statutory documents that make up the local spatial planning programme (*Örtliches Raumordnungsprogramm*): a strategic development concept (*Örtliches Entwicklungskonzept*) and a comprehensive land use plan (*Flächenwidmungsplan*), while the latter shall comply with the objectives of the former. A third, optional planning instrument is a detailed building plan (*Bebauungsplan*) (Land Niederoesterreich undated a).

Next to land use planning, an Austrian municipality is in charge of financing many services of general interest for their local population, such as local public transport, waste collection, services for children and the elderly and schooling, primary healthcare, etc. (Gruber et al. 2015). For these duties, a municipality can raise local fees for infrastructural services and receives a budget through the re-distribution system of taxes collected at the federal and national state levels (e.g. VAT, income tax, etc.) The redistribution mechanism follows the number of inhabitants (Fassmann and Humer 2013).

### Assessment tool: the Lower Austrian infrastructural cost calculator (NIKK)

In regarding service provision and land use planning as interconnected policy tasks (Gruber et al. 2017; Humer 2014), the regional planning authority of the federal state of Lower Austria developed an expert planning tool (*Niederösterreichischer Infrastrukturkostenkalkulator*—*NIKK*) (Land Niederoesterreich undated b).

The planning instrument NIKK (Land Niederoesterreich undated b) is a freely accessible online tool and is meant to support local decision-makers when preparing for settlement enlargement and estimating the financial consequences of infrastructural costs related to changes in the number of inhabitants and the construction of various housing types. On the expenses side, costs for constructing and maintaining technical and network infrastructure (such as streets, electricity lines etc.) as well as accruing costs for social services (such as kindergarten, primary schooling, public playgrounds etc.) are listed separately. On the income side, revenues from increased tax redistribution and additional local infrastructure fees paid by inhabitants are taken into account. Various time horizons can be selected, and additional background information is provided by the federal state—such as a localised population prognosis—can be integrated into the application. The standard application is prepared as an open access web interface with six thematic windows for inserting values for (1) settlement types, (2) technical infrastructure, (3) housing type proportions, (4) land tax, (5) social infrastructure, and (6) development costs. The calculation model behind the application is informed and authorised by the competent sector offices of the federal state, in consultation with experts. The idea of the tool as such would be transferable beyond Lower Austria but the data in the background build on real experiences, or, legal regulations in place, and represent a place-based context of a respective municipality in Lower Austria.

### Case study area: new high-speed railway station on the green field follows new residential area

**Fig. 1:**
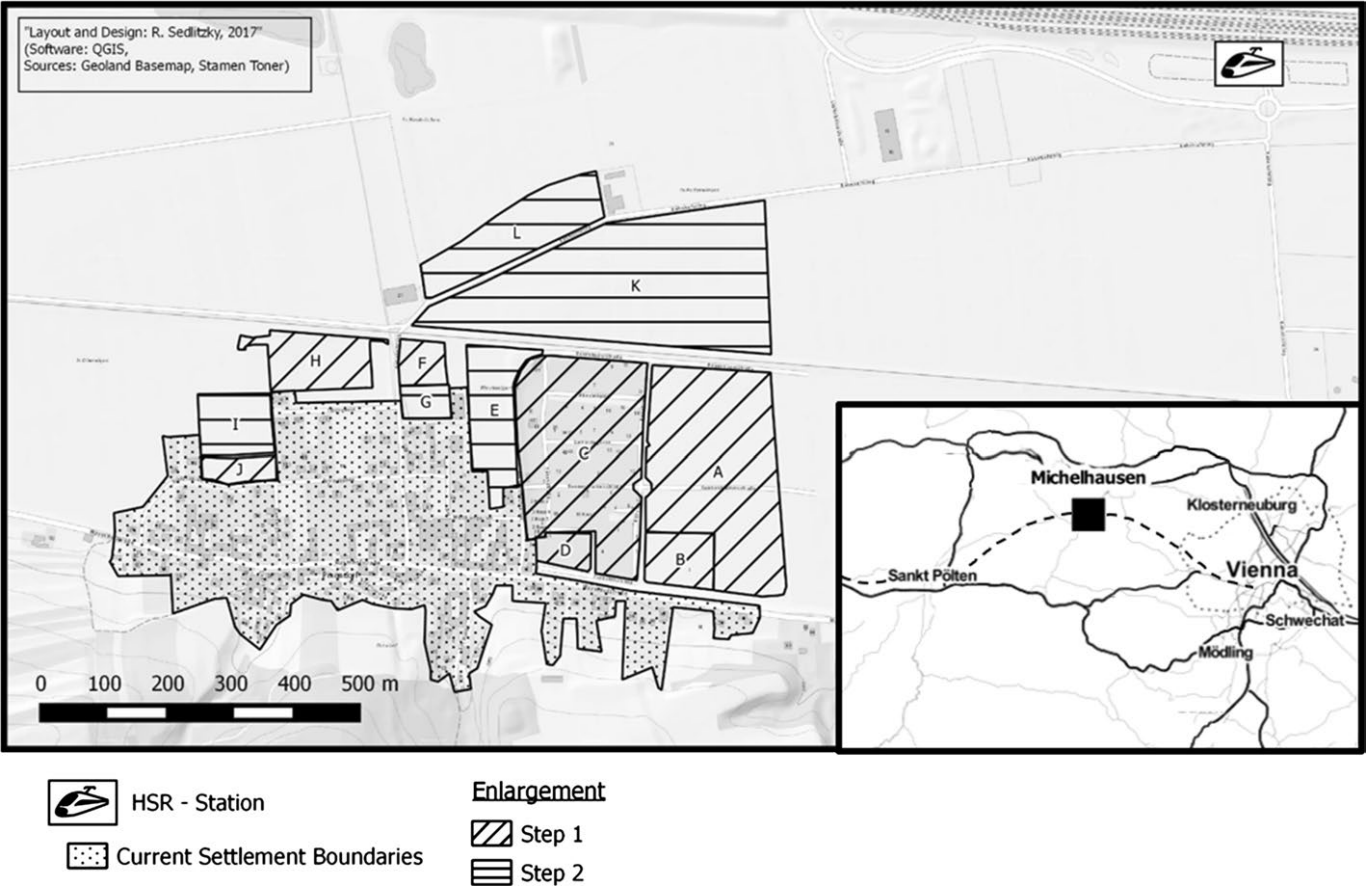
Newly designated and future potential building land plots in Pixendorf/Michelhausen

Michelhausen is—according to Statistik Austria’s urban–rural typology (Statistik Austria undated b)—a ‘centrally located rural municipality’ in the region of Tullnerfeld in the federal state of Lower Austria, halfway between the federal capital St. Pölten and the national capital Vienna (see overview map in Fig. 1). Its population development has grown consistently but slowly over the last decades. Together with the Danube to the north, and the Vienna Woods—a UNESCO biosphere reserve—to the east, there were major accessibility obstacles for the Tullnerfeld that excluded it from the greater suburbanisation processes that otherwise took place around Vienna (Fassmann et al. 2009; Helbich and Leitner 2009; Musil and Pindur 2008). Recently, a major infrastructure project appeared as a potential ‘game changer’ that allowed for a regional re-configuration (Musil 2012, p. 3f). As parts of the improvements to the European TEN-Corridor Rhine-Danube, a new high-speed railway (HSR) section between St. Pölten and Vienna was constructed, including an additional station (*Bahnhof Tullnerfeld*), practically speaking, ‘on the green field’ in-between a couple of rural municipalities of the Tullnerfeld, one of which is Michelhausen. Despite this unconventional location of a HSR station, it dramatically improved the accessibility of Michelhausen. The municipality is now in less than 30 min commuter time distance to Vienna, which has created an opportunity for attracting private real estate investment.^3^ This opened a development path for Michelhausen from a so far—to use Kunzmann’s (2010) terminology—small rural municipality in the metropolitan periphery into a city-regionally integrated suburban area.

Despite the huge infrastructure project plans, the region was lacking a regional planning perspective from top-down and there was no shared commitment to a coordinated inter-municipal strategy from side of the three municipalities bordering the new HSR station. In response to the HSR station, the municipality of Langenrohr developed plans for using the yet unbuilt land to the North of the HSR station for industrial purposes. The municipality to the East, Judenau-Baumgarten, opted for not promoting any building activities near the HSR station but conserve its very rural character. Alike, Michelhausen, located to the West and South of the HSR station, singularly updated its local strategic development concept in 2015, setting out a strategic objective to rapidly increase the number of inhabitants by roughly 25% within 3 years—from around 2800 to 3500. The newly gained upgrade in city-regional accessibility shall follow growth, enabled by new residential areas. The historically grown settlements of the municipality were out of the question when it came to accommodating additional inhabitants through densification or rebuilding measures. Instead, development should concentrate on enlarging the current boundaries of Pixendorf, a settlement of around 300 inhabitants with a classically rural, detached structure in the North-East of Michelhausen, towards the newly opened HSR station. The respective, privately owned plots were assigned ‘building land for housing purpose’ in the land use plan—without any further detail on the type or density of housing in an eventual detailed building plan. A private real-estate company bought up, parcelled, sold and partly developed the new residential area labelled as *Wohnpark Tullnerfeld*. The residential area comprised around 80 plots of land for single-family housing, sized ca. 450–750 m^2^ and one multi-storey house with 37 flats, 45–105 m^2^. By end of 2017, almost all plots (with a rising price/m^2^ of 130 EUR in 2013 to 150 EUR in 2016) and flats (price/m^2^ varying around 2500 EUR) have been sold (Wohnpark Tullnerfeld undated). Evidently, *Wohnpark Tullnerfeld* precisely represents the principle components of urban sprawl (Hamidi et al. 2015), mono-functional, low-density land development.

### Method and results: alternatives of settlement enlargement and housing types

We employ the planning instrument NIKK to create various alternatives of settlement development and related costs—with differing size of enlargement areas as well as different proportions of housing types. We back the assumptions in the five alternatives by a GIS-supported analysis of the municipal territory’s potential for further building land, as well as a thorough document review of the municipality’s strategic development concept and the comprehensive land use plan. Additionally, we carried out three expert interviews with current and former members of the federal planning authority of Lower Austria in the different phases of the study for re-examining the key assumptions of the alternatives, for correctly applying the planning tool NIKK, and for contextualising results.^4^

What concerns the case, the situation as such sounds fair from a local spatial planning perspective. Firstly, there is the strategic objective of population growth in the strategic development concept, envisaging an increase of 700 inhabitants, reasoned by a major improvement of regional accessibility (HSR station). Secondly, there is new designation for housing in the land use plan, enlarging an existing settlement area close to the new HSR station. Thirdly, there is a real estate developer that mobilises the building land, triggering serious demand from private owners on the free market. If the envisaged growth is profitable tough for the municipality—and under which built environmental conditions—will be assessed in the following calculations by applying the federal planning tool NIKK (as introduced in Sect. 3.2). In total, five alternative settlement developments and related public financial consequences are presented. A first alternative follows the objectives in the planning documents of Michelhausen, while the nextfour alternatives alter to that in terms of population size and/or land consumption; see Table 1 for accurate designations of housing types to each plot per alternative.

**Table 1:** Housing type per plot and alternative

A–K plots as assigned in Fig. 1	Alternative 1	Alternative 2	Alternative 3	Alternative 4	Alternative 5
Single-family houses	A, C, F, H, J	C		A, B, I, K, L	
Row houses		A, F, H, J	A, C	E, H	A, C, E, K, L
Multi-storey houses	B, D	B, D	B, D, F, H, J	B, D, G, F, J	B, D, F, G–J

The new building land for the residential area is located in-between the existing settlement area of Pixendorf and the new HSR station (see plots A–D in Fig. 1). Those plots plus three more (plots F, H, J in Fig. 1) are designated for building land, which amounts to around 15.8 ha—roughly the amount of land consumption in Austria every day. Another possible 13.9 ha of further building land—not yet designated in the land use plan—is illustrated as enlargement step 2 in Fig. 1. Alternative 1 takes up the plans for *Wohnpark Tullnerfeld* and extends the so-far traditional settlement type of detached housing in the step 1 plots. Alternative 1, with over 90% of the area being built up with single-family houses and the remaining area with multi-storey houses, shows that neither the strategic objective of plus 700 inhabitants nor a financial positive effect can be reached; neither in the short term of 5 years nor in the long term over 20 years (see Table 2). By alternatives 2 and 3, an incremental increase of settlement density is tested while staying with the area extension of step 1. Alternative 2 offers a varied built-up area with only one third assigned to single-family houses, more than half of the area with row houses and again just 8.5% multi-storey houses. Alternative 3 suggests an even more compact settlement type; dismissing any detached housing. While all three alternatives result in a financial loss for Michelhausen after 5 years, the intended goal of approximately + 700 inhabitants can be nearly achieved by alternative 3. In the long run over 20 years, the + 700 inhabitants target is not reached, however financially, alternatives 2 and 3 pay off for the municipality, due to less infrastructural costs deriving from a more efficient and dense type of housing. Financial details on technical and social infrastructure expenses and fees as well as tax-revenues are given in Table 3 for every alternative. Comparing the most compact housing type, which is alternative 3, to the traditional and envisaged housing type, which is alternative 1, unfolds that the infrastructure expenses are quite the same, while alternative 3 could host over 60% more inhabitants (n = 696) than alternative 1 (n = 426) could do.

**Table 2:** Characteristics of the five alternatives and main outcomes

	Alternative 1	Alternative 2	Alternative 3	Alternative 4	Alternative 5
*Type of housing*	Detached	Varied	Compact	Detached	Compact
Single-family houses	91.5%	36.4%		80.4%	
Row houses		55.1%	76.0%	10.5%	82.0%
Multi-storey houses	8.5%	8.5%	24.0%	9.1%	18.0%
*Enlargement area (in ha)*					
Total	15.8	15.8	15.8	29.7	29.7
Short term perspective	15.8	15.8	15.8	15.8	15.8
Long term perspective	n.a.	n.a.	n.a.	13.9	13.9
*New inhabitants (no.)*					
After 5 years	426	577	696	n.a.	n.a.
After 20 years	560	595	637	1.053	1.182
*Financial balance (in Mill. €)*					
After 5 years	− 1.93	− 0.96	− 1.05	n.a.	n.a.
After 20 years	− 0.07	1.33	1.82	− 1.00	2.38

**Table 3:** Public financial details of the five alternatives

In Mill. €	Tax re-distribution	Social infrastructure	Technical infrastructure	Sum
Alternative 1 detached				
After 5 years				
Expenses	0.191	0.278	3.973	4.442
Revenues	0.336		2.180	2.515
Balance	0.145	− 0.278	− 1.793	− 1.926
After 20 years				
Expenses	2.781	1.693	4.745	9.219
Revenues	5.403		3.749	9.151
Balance	2.622	− 1.693	− 0.996	− 0.068
Alternative 2 varied				
After 5 years				
Expenses	0.252	0.349	3.958	4.560
Revenues	0.448		3.153	3.601
Balance	0.196	− 0.349	− 0.805	− 0.958
After 20 years				
Expenses	3.406	1.648	4.730	9.783
Revenues	6.632		4.482	11.114
Balance	3.226	− 1.648	− 0.247	1.331
Alternative 3 compact				
After 5 years				
Expenses	0.292	0.373	3.962	4.627
Revenues	0.522		3.051	3.572
Balance	0.230	− 0.373	− 0.911	− 1.054
After 20 years				
Expenses	3.885	1.618	4.734	10.237
Revenues	7.592		4.464	12.056
Balance	3.707	− 1.618	− 0.270	1.819
Alternative 4 detached				
After 20 years				
Expenses	4.592	2.666	9.166	16.674
Revenues	8.869		6.802	15.671
Balance	4.277	− 2.666	− 2.364	− 1.003
Alternative 5 compact				
After 20 years				
Expenses	6.076	2.435	9.090	17.601
Revenues	11.769		8.212	19.981
Balance	5.693	− 2.435	− 0.879	2.380

If the aim of + 700 inhabitants is to be reached/exceeded, more land, denser housing and more time is necessary. Therefore, alternatives 4 and 5 include further potential building land (see step 2 plots in Fig. 1) in the calculation, offering again a detached (alternative 4) versus a compact (alternative 5) type of settlement structure. Since the additional land is not yet confirmed in the municipality’s land use plan, alternatives 4 and 5 are only provided for a long-term perspective of 20 years. In both cases, the number of inhabitants will be more than + 1000, however financially the consequences for the municipality differ significantly. The compact settlement type (alternative 5) brings a positive balance of around €2.4 million to the local budget, a continued traditional, detached housing type (alternative 4) would harm the local budget by around €1 million.

### Discussion: long time horizons and dense settlement types pay off

In this section, we will prepare arguments and finally answer the stated research question to the case study: ‘What short-term and long-term financial impact does it have on a municipality, where and how new inhabitants are being settled?’ From the results across the five alternatives, a clear picture develops that only settlement types of a certain density result in a financial gain for the municipality. In a short-term perspective over 5 years, the aim of a population increase—that was set in the strategic development concept for an even shorter period of 3 years—proves unrealistic, even in a new compact settlement. Growing in a more substantial way—in this case by including all further potential building land (including step 2 plots in Fig. 1) into the development—can be profitable for the municipality if row houses or multi-storey houses are chosen as main types but in any case, this takes long time horizons and high financial turnovers. Important to say is that the overall public expenses of alternatives 4 and 5 (€16–17 million) are much higher than for the smaller scale alternatives 1 to 3 (€9–10 million). So the financial management and turnover—including pre-financing and eventual bank loans—in the extended alternatives could become very challenging for Michelhausen’s public budget; see expenses after 20 years for each alternative given in the last column of Table 3.

This assessment of local planning objectives has not included alternatives based on inner-development or the densification of existing settlements, as they were not considered viable according to Michelhausen’s strategic development concept. Had they have been, a major portion of the expenses for new technical infrastructural development falls away as existing settlements are presumed to already be equipped with necessary street and network infrastructures, while the type of settlement development is less directly affecting social infrastructure development. Generally, little difference is expected for the social infrastructure part of the calculation whether the increase of inhabitants takes place through settlement enlargement or inner densification. More inhabitants in a municipality means higher total costs for social infrastructure, which—to make matters complicated—do not increase in a linear fashion, but are linked to some cohort effects and thresholds of population figures. Depending on the type of housing—small flat or detached house—population will be of different age and household structures. We briefly illustrate this combined circumstance using the example of the expected new inhabitants of alternative 2 (see Table 4). If a certain threshold of new inhabitants of the kindergarten age group is reached, the municipality is obliged to open an extra class in the municipal kindergarten. In alternative 2 (Table 4), the number of kindergarten children increases from four in year 1 to over 50 in year 4 which would require at least two additional classes in the local kindergarten. A peak in year 7 with over 70 kindergarten children—and maybe three additional classes—would follow a permanent decrease of this cohort effect down to a rather stable amount of 30 children after around 15 years with the effects passing on to primary schooling and later secondary schooling.

**Table 4:** Yearly population development according to alternative 2

Year	1	2	3	4	5	6	7	8	9	10
Kindergarten age	4	22	36	57	67	68	72	52	49	48
Primary school age	7	29	39	46	55	52	61	54	54	44
Total population	89	225	341	474	578	576	605	622	584	578

Year	11	12	13	14	15	16	17	18	19	20
Kindergarten age	38	36	41	34	32	34	30	28	33	31
Primary school age	44	44	45	30	30	28	29	26	24	22
Total population	590	559	557	556	563	576	586	588	599	595

We sum up, by answering the research question, yes, there are significant financial repercussions for municipalities based on where and how new inhabitants are settled and the effect on the local public budget over time cannot be underestimated. The empirical exercise confirmed that—from a financial point of view at least—preference should be given to inner-development over settlement enlargement, to compact settlements over detached housing and—regardless of the form of the planned development—pre-financing and negative local public financial balances must be expected in the short term. Only in the long term does efficient planning for growth pay off financially for a municipality. It may be unsurprising findings but some that have to be told with empirical backing, as provided by this short study, in order to slowly increase awareness over potential risks of an undifferentiated planning culture for growth, especially among small, rural-to-suburban municipalities. Finally, the following conclusion contains some learnings made from applying the strategic planning tool NIKK, considers some possible improvements thereof and reflects on local planning practices for growth, at least in the context of Lower Austria.

## Conclusion: assessment of the infrastructural cost calculator and considerations for policy and planning practice

The following considerations focus on the actual planning instruments and procedures in the presented case of Michelhausen and Lower Austria. The planning support tool NIKK provided by the federal state of Lower Austria to their municipalities is a valuable instrument to check the financial consequences of a planned development strategy. The instrument can potentially challenge the predominant, undifferentiated planning culture for growth ‘at all costs’ and raise awareness among local politicians and planners. An extended application in course of this study has revealed some potential for improvement, stemming from current technical and procedural shortcomings. Technically, the tool is by now made to assess settlement enlargements, but it does not address densifications or the filling-in of single empty spots and it is promoted foremost to assess one specific land use development option, and not a series of alternatives in parallel. A first consideration is to enhance such a supportive planning tool with GIS/cartographic features to allow for a place sensitive application and to explicitly show different consequences for inner settlement development versus enlargement.^5^ A second consideration is to enhance the tool for easier building of several alternatives, by, for example, displaying financial results comparatively at once. Procedurally, the tool is currently optional for municipalities. A third consideration is to include this application into the Lower Austrian planning law and make it an obligatory part of the local statutory planning procedure—likewise a ‘territorial-financial impact assessment’. Behind this recommendation is that local planning instruments yet weakly interlink with each other. An argumentative link between the strategic development concept and the comprehensive land use plan should be formally requested; i.e. specifically locating strategic objectives—such as the ‘+ 700 inhabitants’ objective of Michelhausen—in suitable land use zones. For larger projects, detailed building plans should be issued in smaller, rural settlements too, not only in urban core zones. The planning tool NIKK could procedurally assure a better link.

Reflecting the case study, it becomes apparent that growth (in terms of population and settlement) requires enhanced professionalism of municipal planning to address complex challenges and not least high financial capacities of municipalities due to pre-financing of infrastructural investments. From the side of the federal planning authority, it requires reliable state support tools, such as planning-wise the NIKK or financing-wise state budget funds for loans and pre-financing support. Those state budget funds can be linked to efficient results from planning support tools; e.g. state loans in case of compact settlement development only. Further, growth requests planning with sufficient time horizons, taking into account also population cohort and step-wise settlement densification/enlargement and lastly but crucially, doing planning by thinking in alternatives.

## Footnotes

1 Clearly, several societal and political rationales, not just financial ones, drive the aim for a growing local community. However, the study will be concise by focusing on the public financial consequences of a municipality, which is the scope of the planning tool to be tested. The study consequently does not look at possible wider, indirect private financial or non-financial aspects of local development that come with an increase in the number of inhabitants, until for example, eventual increase of local social capital and diversity, or other societal benefits. Likewise, the study will not go into detail of further costs of (sprawled) settlement development than local public ones. See Ewing and Hamidi (2015, p. 418ff) for a comprehensive discussion of the costs of sprawl in terms of transport, congestion, as well as public health.

2 While the national level has no formal role at all, the nine federal states of Austria pass separate spatial planning laws and function as a control body for changes and amendments of local planning documents. See further literature on the exceptionally federalised Austrian spatial planning system for example here: Faludi (1998), Fassmann and Humer (2013) and Humer (2018a, b).

3 Delage (2016) finds evidence of how a high-speed railway station can become a trigger for private investment, in her case in the shrinking city of Saint-Étienne. This work was part of a special issue that highlights the wider—also financial—costs and effects of high-speed railway and station construction for local development and tourism (Delaplace and Dobruszkes 2016).

4 The three semi-structured face-to-face interviews were conducted in May 2016. Acknowledgements for their participation, time and expertise go to the interviewees H. Hamader, K. Pelz-Grundner and M. Maxian.

5 Another consideration that exceeds the scope of this study but is nevertheless worth sharing, is to possibly modify the NIKK tool to be also used for municipalities threatened by ongoing population shrinking, accompanied with high shares of housing vacancies and oversized infrastructure, in order to support those in decisions of deconstructing or consolidating settlement areas.
